# Gelatin–Polyvinyl Alcohol Microspheres for Controlled and Sustained Release of BMP-2 and VEGF Enhance Osteogenic and Angiogenic Cell Differentiation

**DOI:** 10.3390/gels12040326

**Published:** 2026-04-11

**Authors:** Varvara Platania, Konstantinos Loukelis, Maria Chatzinikolaidou

**Affiliations:** 1 Department of Materials Science and Engineering, University of Crete, 70013 Heraklion, Greece; vplatania@materials.uoc.gr (V.P.);; 2 Institute of Electronic Structure and Laser (IESL), Foundation for Research and Technology Hellas (FORTH), 70013 Heraklion, Greece

**Keywords:** bone morphogenetic protein 2, vascular endothelial growth factor, angiogenesis, osteogenesis, drug delivery, growth factors

## Abstract

Bone morphogenetic protein-2 (BMP-2) and vascular endothelial growth factor (VEGF) play a pivotal role in promoting osteogenesis and angiogenesis that concurrently take place during bone regeneration. The rapid degradation and diffusion of these growth factors, combined with the potential side effects associated with their exogenous insertion, limit their applications. To overcome these shortcomings, we developed a controlled release system for BMP-2 and VEGF on microspheres comprising gelatin (Gel) and polyvinyl alcohol (PVA). We fabricated Gel–PVA microspheres using a constant Gel concentration of 10% *w*/*v* and a varied PVA concentration of 0, 5, and 10% *w*/*v* (Gel–PVA0%, Gel–PVA5%, and Gel–PVA10%, respectively). The microspheres were loaded with the model protein bovine serum albumin (BSA) first. The Gel–PVA10% microspheres demonstrated significantly higher loading capacity and encapsulation efficiency, as well as lower cumulative release rate, compared to the Gel–PVA5% ones when loaded with BSA. Thus, the microspheres with the Gel–PVA10% composition were selected for loading with BMP-2 and VEGF. Kinetic studies of BMP-2 and VEGF loaded into Gel–PVA10% microspheres indicated similar results to those with BSA. The microsphere concentration with the optimal cytocompatibility was 0.5 mg/mL, and it was applied for the assessment of the osteogenic differentiation using bone marrow-derived mesenchymal stem cells (MSCs), and for the angiogenic differentiation in Wharton jelly and adipose-derived MSCs. Alkaline phosphatase activity, collagen secretion, and calcium mineralization were significantly upregulated in the presence of BMP-2-loaded microspheres, while tubular formation and PECAM-1 secretion were significantly higher in VEGF-loaded microspheres compared to the unloaded control, demonstrating their effectiveness as drug delivery carriers.

## 1. Introduction

Bone tissue engineering offers new therapeutic avenues, including scaffolds, microspheres, and other biocompatible constructs, which in turn allow for cell survival, adhesion, proliferation, and differentiation. The utilization of bioactive molecules such as growth factors (GFs), which can act as regulators of the differentiation of various stem cell lines towards specific lineages, presents a potent strategy for accelerated tissue regeneration. Bone morphogenetic protein 2 (BMP-2) is a thoroughly investigated growth factor, with one of its main metabolic functions being the osteoblastic differentiation process of mesenchymal stem cells (MSCs) [1]. Additionally, vascular endothelial growth factor (VEGF) has also been found to depict a strong correlation with osteogenesis, acting as one of the key mediators of neovascularization for the newly regenerated bone tissue [2]. Therefore, the development of carriers that can enable the concurrent delivery of both growth factors following a controlled release pattern presents great interest, due to their synergistic activity in bone reconstitution [3,4].

A significant challenge in utilizing growth factors for bone regeneration is their rapid degradation profiles outside the body, requiring high exogenous doses to counter this, a scenario that is both impractical and economically non-viable [5]. Additionally, high levels of growth factors can elicit uncontrolled side effects locally, including hyperpermeability of the blood vessels, anatomical abnormalities that can hinder the physiological activity of the newly formed tissue, and, in some cases, tumor growth [6]. To address these challenges, a drug delivery system with tunable sustainability and controlled protein release profiles is essential to allow desorption of the growth factors at controllable rates. On that note, multiple delivery systems have been developed, focusing primarily on polymeric microspheres [7], nanospheres [8], liposomes [9], and nanofibers [10], as well as hydrogels [11] and their equivalent combinations, such as drug-loaded hybrid hydrogel microspheres. Among these systems, the encapsulation of growth factors in hybrid hydrogel microspheres is a favorable technique, as their release rates are directly tied to their geometrical characteristics and chemical composition [12].

A range of studies have explored the potential of gelatin-based and polyvinyl alcohol (PVA)-based microspheres as drug delivery carriers [13,14]. Gelatin is a product of native extracellular matrix (ECM) collagen hydrolysis. It is a biocompatible and inexpensive protein, which has been investigated for the preparation of controlled release systems. Due to these properties, gelatin is widely employed for the fabrication of microspheres as drug and gene delivery platforms [14]. Gelatin and related protein-based carriers continue to attract significant interest for controlled protein delivery due to their biocompatibility, biodegradability, and tunable release behavior, as extensively reviewed in the recent literature on gelatin-based drug delivery systems [15]. PVA is a hydrophilic polymer rich in hydroxyl groups that is capable of forming extensive intermolecular hydrogen bonds with gelatin chains, leading to the formation of physically crosslinked networks [16]. These supramolecular interactions, together with polymer chain entanglement, promote the formation of denser three-dimensional structures with reduced mesh size and enhanced mechanical stability [17]. Such physically crosslinked networks have been shown to significantly affect diffusion behavior, swelling, and degradation kinetics in hydrogel-based drug delivery systems.

Recent work highlights how key formulation parameters, such as polymer composition and crosslinking density, affect degradation and release behavior in gelatin microspheres, enabling tailored delivery profiles under mild processing conditions [18]. In addition, studies on gelatin microspheres prepared with alternative crosslinkers demonstrate controlled release of hydrophilic drugs and the potential for integrating these carriers into composite systems [19]. However, their applicability is weighed down by some limitations. One of the primary concerns is the thermal instability of gelatin microspheres at temperatures close to 37 °C, which can result in rapid degradation and thus premature release of the contents of gelatin-containing constructs [20]. This instability may necessitate higher initial doses of growth factors to compensate for the rapid loss of bioactivity, which can be impractical and costly [21]. Hydrogel microspheres composed of natural and synthetic polymers have undergone substantial development, with recent work summarizing advances in fabrication techniques, functionalization, and biomedical application potential across multiple platforms [12]. PVA, another widely utilized biomaterial for tissue engineering applications, is a water-soluble synthetic polymer known for its ability to provide structural stability to hydrogel systems. Therefore, incorporating PVA in gelatin-based systems in varying concentrations could significantly reduce the diffusion rate of the loaded content, thus achieving optimal biological response [22,23].

The objective of this study was to develop and evaluate a hybrid microsphere-based delivery system composed of gelatin and PVA for the controlled delivery of BMP-2 and VEGF, two key growth factors with synergistic roles in bone regeneration. While gelatin and PVA have been combined in various biomedical applications as drug carriers, their compositional tuning under crosslinking to modulate release kinetics without covalent immobilization of growth factors remains largely unexplored. In this study, we demonstrate that solely varying the PVA fraction within a constant gelatin network crosslinked with glutaraldehyde provides a predictable and scalable strategy to control degradation and protein diffusion, distinguishing this system from chemically functionalized or ligand-conjugated Gel–PVA carriers. Recent reviews further highlight the expanding scope of gelatin microparticle systems, including regenerative medicine strategies and drug screening applications, underscoring the broad utility of these carriers in biomedical research [24]. The tunable release kinetics achieved by varying the PVA concentration, allowing for sustained delivery without covalent conjugation of the two growth factors, present an innovative feature of this work.

Furthermore, the bioactivity of the released factors from the microspheres was validated using bone marrow-derived (BM) mesenchymal stem cells (MSCs) for osteogenic responses, and Wharton jelly (WJ) and adipose-derived (AD) MSCs to monitor angiogenic responses. BM-MSCs were selected for the osteogenic assays in this study since they have been extensively characterized for high osteogenic differentiation potential and have been shown to express key osteogenic markers earlier and more robustly than other MSC sources, making them a preferred model for bone formation studies [25]. Moreover, for angiogenic differentiation assays, MSC populations such as WJ-MSCs or AD-MSCs are acknowledged to have enhanced angiogenic paracrine activity and endothelial responsiveness, supporting their suitability for studies related to VEGF delivery [26]. This dual-functional, MSC-compatible system may offer a strategy for enhancing vascularized bone tissue regeneration.

## 2. Results

### 2.1. Physicochemical Characterization of Gel–PVA Microspheres

#### 2.1.1. Morphology and Size Distribution of Gel–PVA Microspheres

**Figure 1:**
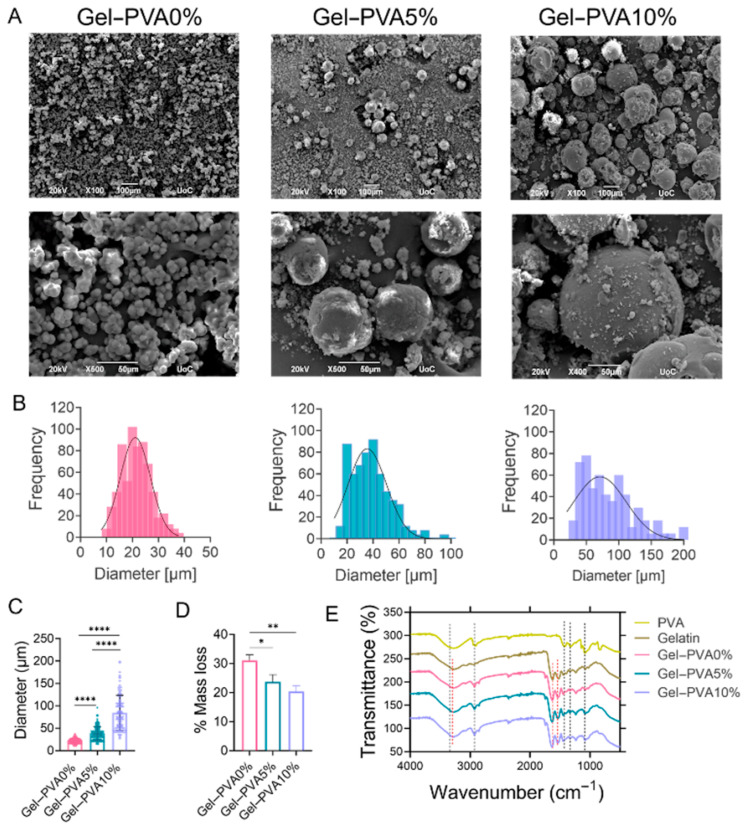
Representative SEM images and physicochemical characterization of microspheres. Low-magnification SEM micrographs (magnification of ×100) used for particle size analysis (**A**) **upper panel**; higher-magnification views (magnification of ×400–500) illustrating microsphere surface morphology (**A**) **lower panel**. The scale bars represent 100 μm and 50 μm, respectively. Histograms showing the number of microspheres expressed as a frequency within each diameter range fitted with a Gaussian curve representing the size distribution (**B**). Average diameters of microspheres. The values represent the means ± standard deviation, with each type based on over 400 measurements (**C**). Evaluation of microsphere degradation after 48 h (**D**). FTIR spectra of different microsphere types and their base components: gelatin and PVA (**E**). Error bars represent the standard deviation (SD) of all replicates from three independent experiments (* *p* < 0.05, ** *p* < 0.01, and **** *p* < 0.0001 denote a statistically significant difference compared to the Gel–PVA0% control). Color coding: pink corresponds to Gel–PVA0%, green to Gel–PVA5%, and purple to Gel–PVA10%.

SEM images of gelatin microspheres with varying concentrations of PVA (0%, 5%, and 10% *w*/*v*) at two magnifications, illustrating their size, structure, and morphology, are presented in Figure 1A. In each composition, uniformly shaped spherical particles are evident, with diameters increasing proportionally to the concentration of PVA. To represent the size distribution of these fabricated microspheres, a frequency histogram analysis was conducted, with a bar graph with the *x*-axis indicating particle size and the *y*-axis representing the relative count of microspheres at each concentration level. The resulting histograms, accompanied by Gaussian curves, are depicted in Figure 1B. These distributions reveal that all compositions led to particles within a size range of 10 to 200 μm. Higher PVA concentrations led to the formation of larger microspheres, with sizes ranging between 10 and 40 μm for Gel–PVA0%, 20 and 80 μm for Gel–PVA5%, and 40 and 200 μm for Gel–PVA10%. Figure 1C summarizes the average diameter comparison of the three compositions.

#### 2.1.2. Physicochemical Characterization of the Microspheres

The degradation rate profiles of the microspheres are presented in Figure 1D. Gel–PVA0% showed a mass loss equal to 31 ± 2%, with the incremental increase in PVA concentration leading to the expectedly lower values of 24 ± 3% (Gel–PVA5%) and 20 ± 2% (Gel–PVA10%), respectively. FTIR spectra analysis was performed for all the microsphere types crosslinked with 0.025% *v*/*v* glutaraldehyde (Figure 1E). Pure gelatin showed a stretch at 3200–3500 cm^−1^ due to the presence of N-H bonds as well as O-H bonds that can be found in the same region. Moreover, there was an intense peak at 1650 cm^−1^, which is representative of the presence of C=O bonds. The main peaks of PVA were observed at 3280, 2917, 1690, 1425, 1324, 1081, and 839 cm^−1^. These peaks can be ascribed to the O–H stretching vibration of the hydroxy groups, CH asymmetric stretching vibration, C=O carbonyl stretch, C–H bending vibration of CH_2_, C–H deformation vibration, C–O stretching of acetyl groups, and C-C stretching vibration, accordingly [27]. All produced microsphere compositions retained the aforementioned peaks and stretches from their base constituents.

#### 2.1.3. In Vitro Protein Absorption and Release Study with BSA as the Model Protein

**Figure 2:**
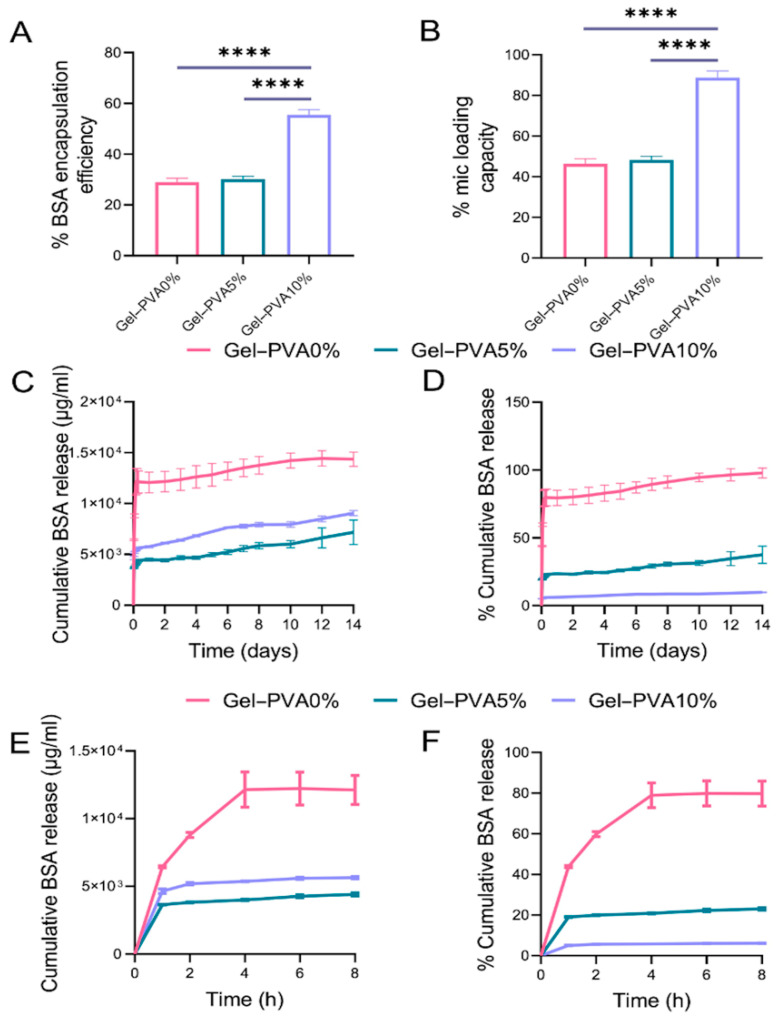
In vitro protein absorption and release using BSA as the model protein. Evaluation of % of encapsulation efficiency (**A**), % of microsphere loading capacity (**B**), released protein concentration (**C**), and the corresponding % of cumulative release (**D**) from the microspheres. Magnified representation of the early-stage release profiles at 0–8 h from Gel–PVA0%, Gel–PVA5%, and Gel–PVA10% microspheres expressed in concentration (μg/mL) (**E**) and % of the released BSA (**F**). Error bars represent the standard deviation (SD) of three replicates from three independent experiments (**** *p* < 0.0001 denotes a statistically significant difference compared to the Gel–PVA0% control).

The percentage of BSA that was successfully entrapped into the microspheres was calculated, aiming at the determination of %EE (Figure 2A). All concentrations exhibited high values, with the minimum BSA encapsulation percentage belonging to the Gel–PVA10% composition being up to 80%, and the maximum levels of 98% EE belonging to Gel–PVA0% microspheres, with the latter results being in agreement with previous studies [28].

In order to investigate the effect of PVA concentration on the in vitro protein absorption of the Gel–PVA0%, Gel–PVA5%, and Gel–PVA10% microspheres, the passive% loading capacity (LC) of the BSA was examined (Figure 2B). The Gel–PVA10% loading capacity exceeded the other two microsphere types significantly, displaying the highest loaded amount of 85% per unit weight of microspheres, followed by 46% and 43% in the case of Gel–PVA5% and Gel–PVA0%, respectively.

The release profiles of BSA from the different Gel–PVA microspheres are illustrated in Figure 2C,D. The initial burst release phase occurs during the first 24 h, switching to a relatively steady release rate of the protein for the next 14 days. The Gel–PVA0% control microspheres demonstrated the fastest release kinetics out of all formulations, with a burst release of almost 80% of the total encapsulated protein during the first 24 h. The Gel–PVA5% microspheres displayed a burst release of approximately 20% of the total encapsulated protein after 24 h and 35% release during the first two weeks. Finally, an increase in PVA concentration up to 10% led to an even slower protein release profile, peaking at less than 10% of the total protein mass during the 14-day period. As shown in the magnified early time plots of 0–8 h (Figure 2E,F), Gel–PVA0% microspheres exhibited a pronounced burst release, whereas increasing PVA content markedly suppressed early release, with Gel–PVA10% showing minimal release (<7%) during this period.

### 2.2. In Vitro Biocompatibility of Gel–PVA Microspheres

**Figure 3:**
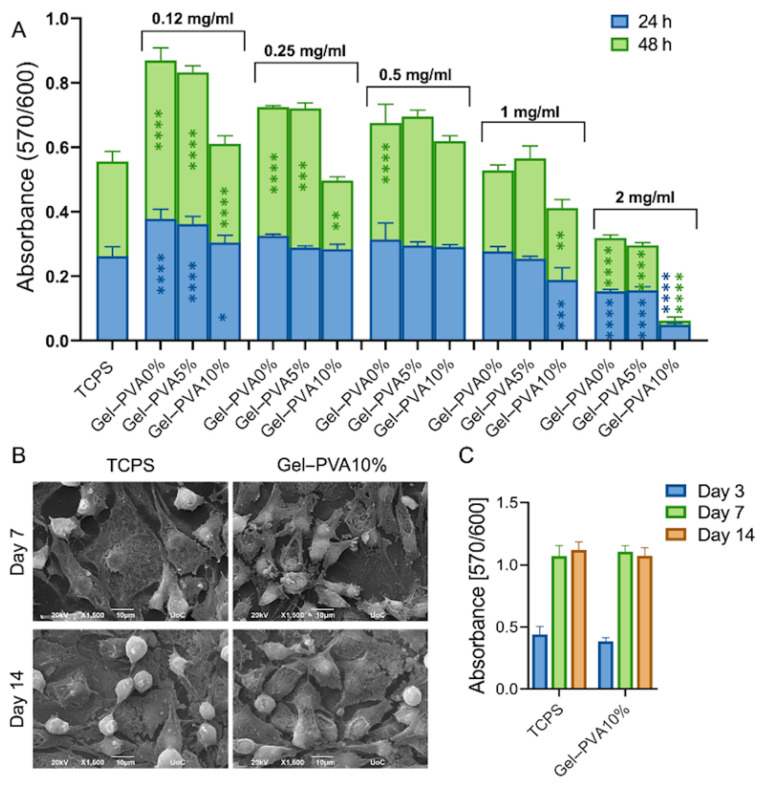
Cell viability assessment in the presence of Gel–PVA0%, Gel–PVA5%, and Gel–PVA10% microspheres at concentrations of 0.125, 0.25, 0.5, 1, and 2 mg/mL at 24 and 48 h in culture (**A**). SEM images depict the cellular adhesion and morphology of the selected sample composition Gel–PVA10% at a concentration of 0.5 mg/mL at days 7 and 14, and compared with TCPS (**B**); cell viability in the presence of 0.5 mg/mL Gel–PVA10% microspheres at days 3, 7, and 14 (**C**). Scale bar represents 10 μm. Error bars represent the standard deviation of three replicates from three independent experiments (* *p* < 0.05, ** *p* < 0.01, *** *p* < 0.001, and **** *p* < 0.0001 denote a statistically significant difference compared to the TCPS control; the absence of asterisks denotes non-significant differences).

The cell viability was quantified using five different concentrations (0.125, 0.25, 0.5, 1, and 2 mg/mL) of the different Gel–PVA microspheres using L929 murine fibroblasts after 24 and 48 h in culture (Figure 3A). The 2 mg/mL concentration depicted a significant decrease in cell viability, independently of the microsphere composition, compared to the TCPS control, at all time points. Reduction to 1 mg/mL microsphere concentration did not present any profound differences between the TCPS control and the different microsphere compositions for the first 24 and 48 h. All compositions investigated at concentrations of 0.5 mg/mL and lower showed excellent cytocompatibility (Figure 3A). Thus, all further biological experiments were conducted with this particular concentration. The cell adhesion, morphology, and proliferation of L929 murine fibroblasts in the presence of the Gel–PVA10% microspheres at 0.5 mg/mL were performed over a period of 2 weeks. As depicted in Figure 3B, L929 presented excellent adhesion, having adopted their characteristic elongated morphology even by day 7, without any differences being evident between the Gel–PVA10% and TCPS control conditions. Similarly, no variations in biocompatibility were observed among the two samples, with a three-fold cell number increase between days 3 and 7, while retaining similar levels between days 7 and 14 (Figure 3C).

### 2.3. In Vitro BMP-2 and VEGF Absorption and Release Study of the Loaded Gel–PVA10% Microspheres

**Figure 4:**
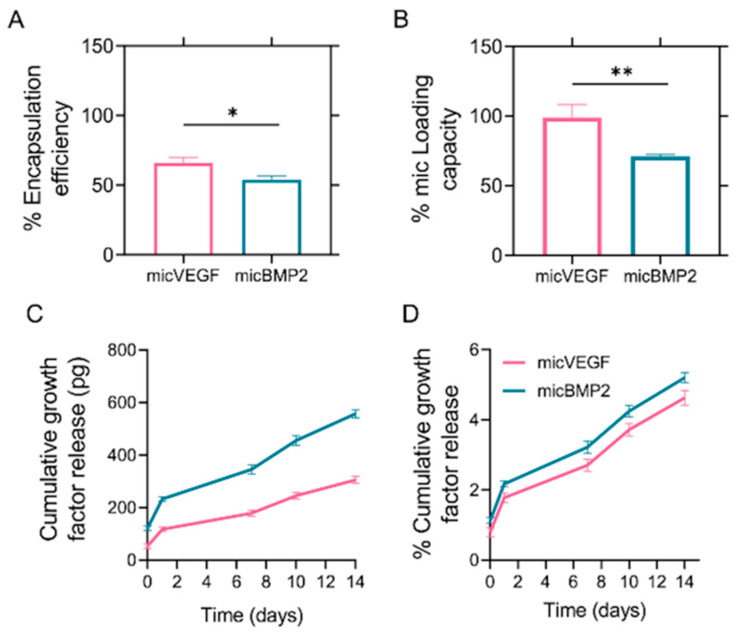
In vitro absorption and release of BMP-2 and VEGF. Evaluation of % of encapsulation efficiency (**A**), % of loading capacity (**B**), released growth factor mass (**C**), and the corresponding % of cumulative growth factor release (**D**) from Gel–PVA10% microspheres. Error bars represent the standard deviation of three replicates from three independent experiments (* *p* < 0.05 and ** *p* < 0.01 denote a statistically significant difference between micVEGF and micBMP2).

The percent encapsulation efficiency (%EE) and loading capacity (%LC) of BMP-2 and VEGF in Gel–PVA10% microspheres were determined to assess the potential of these microspheres for controlled release of growth factors. The %EE for BMP-2 was found to be slightly lower than that for VEGF, with BMP-2 achieving an encapsulation efficiency of approximately 52%, while VEGF exhibited a higher %EE at around 64% (Figure 4A). The loading capacity followed a similar trend, with the Gel–PVA10% microspheres showing a %LC of 68% for BMP-2 and 92% for VEGF, highlighting the capacity of these microspheres to incorporate substantial amounts of both growth factors (Figure 4B).

The release kinetics of BMP-2 and VEGF from the Gel–PVA10% microspheres were monitored over a period of 14 days. The release profiles of both growth factors displayed a characteristic biphasic pattern (Figure 4C,D), similar to that observed with BSA. An initial burst release phase was observed within the first 24 h, followed by a more controlled and sustained release over the subsequent days. For BMP-2, approximately 2.1% of the total encapsulated protein was released during the initial burst phase of the first day. This was followed by a gradual release, reaching a cumulative release of 5% over the 14-day period. VEGF exhibited a slightly higher initial burst release of 1.8%, with a cumulative release of 4.5% by day 14. Compared to the BSA results, the Gel–PVA10% microspheres demonstrated a relatively consistent and slower release profile for both BMP-2 and VEGF. The ability of Gel–PVA10% to modulate the release rates of BMP-2 and VEGF suggests its potential utility as a delivery platform in bone regeneration applications where controlled release is essential for therapeutic efficacy.

For gelatin-based carriers, growth factor uptake is often enhanced at physiological temperature because increased polymer chain mobility and hydrogel swelling improve molecular diffusion and electrostatic interactions. For instance, genipin-crosslinked gelatin microspheres were loaded with BMP-2 at elevated temperature to maximize swelling and uptake efficiency [29]. Conversely, several reports describe growth factor loading under cold conditions and extended incubation to preserve bioactivity during passive adsorption. For instance, BMP-2 and other growth factors have been incubated with microparticles for prolonged periods (e.g., overnight or ≥16 h) at 4 °C to achieve efficient binding while minimizing structural degradation [30].

### 2.4. Osteogenic Response of Human BM-MSCs in the Presence of micBMP2

**Figure 5:**
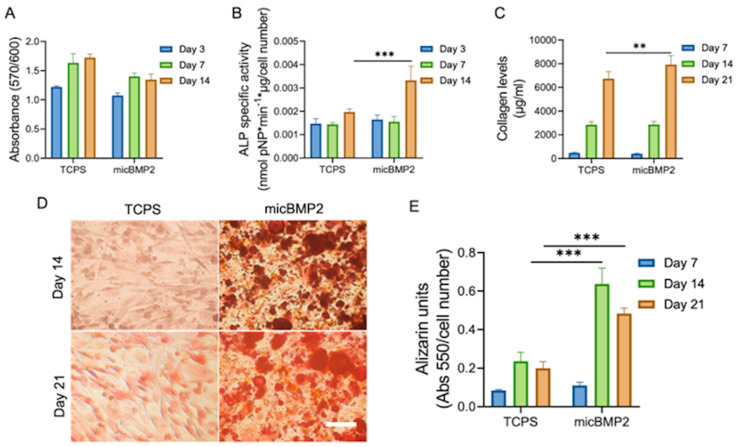
BM-MSC viability and proliferation in the presence of micBMP2 and in the TCPS control (**A**). Osteogenic response of BM-MSCs treated with micBMP2 by means of ALP activity of BM-MSCs normalized by cell number (**B**). Quantification of the produced extracellular collagen by the BM-MSCs (**C**). Alizarin staining of the mineralized calcium (**D**) and determination of normalized calcium produced by BM-MSCs (**E**). Scale bar represents 50 μm. Error bars represent the standard deviation (SD) of three replicates from three independent experiments (** *p* < 0.01 and *** *p* < 0.001 denote a statistically significant difference compared to the TCPS control; the absence of asterisks denotes non-significant differences).

In terms of cell viability, micBMP2 did not significantly affect BM-MSCs throughout the culture period (Figure 5A). The cell numbers increased between days 3 and 7 and remained unchanged by day 14, which is indicative of a shift from their proliferative activity toward differentiation. This behavior is observed during MSC osteogenic commitment, as cells exit the cell cycle and begin expressing lineage-specific markers [31].

To monitor the nature of the systematic promotion of osteogenesis of BM-MSCs in the presence of micBMP2, ALP activity was measured after 3, 7, and 14 days in culture. During days 3 and 7, micBMP2 presence resulted in identical levels of ALP activity with the control TCPS culture. However, a significant enhancement of the enzymatic activity, approximately 1.5-fold compared to the control TCPS culture, was detected after 14 days, as depicted in Figure 5B.

A hallmark of osteoblast differentiation is considered to be the formation of an extracellular matrix. The total collagen secreted in culture supernatants at different time intervals was quantified, as shown in Figure 5C. The significantly higher values of collagen levels after 21 days also indicated an amplified ECM formation by BM-MSCs in the presence of micBMP2.

The calcium mineralization process in the native bone is regarded as a principal marker of the maturation of BM-MSCs to osteoblasts. To validate the calcium levels in the extracellular matrix, Alizarin Red staining was conducted at days 7, 14, and 21. Although both conditions showcased similar levels of calcium ion content at day 7, at day 14, a significant increase was observed, with the micBMP2 condition surpassing the TCPS control by a margin of almost 3-fold. Similarly, between days 14 and 21, the levels of calcium mineralization showed values of 2-fold compared to the TCPS control (Figure 5D,E).

ALP activity, collagen production, and calcium deposition all showed clear upregulation between days 7 and 14, suggesting that while cell proliferation reached a steady state, differentiation processes were actively progressing in response to micBMP2 stimulation.

### 2.5. Angiogenic Response of Human WJ-MSCs and AD-MSCs in the Presence of micVEGF

**Figure 6:**
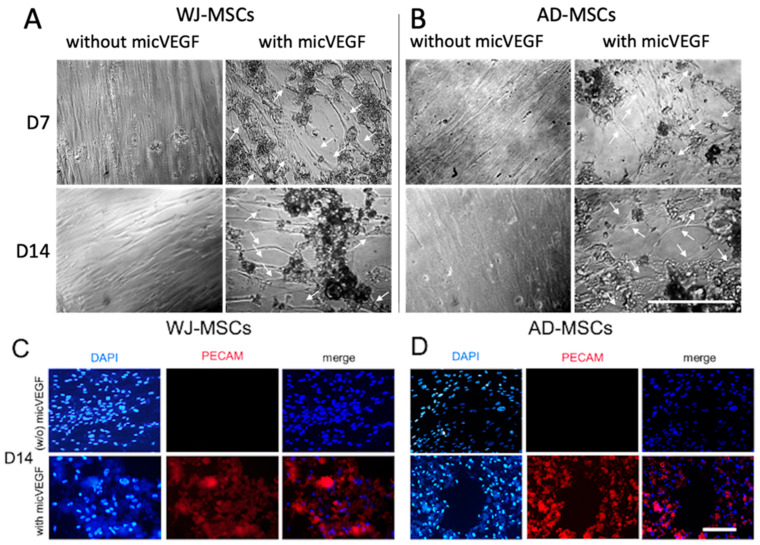
Representative optical microscopy images depicting tubular structure formation of WJ-MSCs (**A**) and AD-MSCs (**B**) in the presence and absence of micVEGF after 7 and 14 days in culture. Scale bar represents 200 μm. White arrows indicate tubular cell organization. Immunofluorescent staining of nuclei stained with DAPI (**left**), endothelial marker PECAM-1 (**middle**), and merged images (**right**) of WJ-MSCs (**C**) and AD-MSCs (**D**) after 14 days in the absence (w/o) and presence of VEGF-loaded Gel–PVA10% microspheres (micVEGF). Scale bar represents 50 μm.

The morphological change in mesenchymal stem cells when guided towards endothelization constitutes one of the most representative markers of an organic vascular network formation. Therefore, the effect of micVEGF on WJ-MSCs and AD-MSCs towards angiogenic differentiation was investigated. Both WJ-MSCs and AD-MSCs maintained their fibroblastic shape when cultured in the absence of micVEGF. Notably, the presence of micVEGF at a concentration of 0.5 mg/mL prompted their transition towards cobblestone morphology (Figure 6), even from the early time point of 7 days. Furthermore, we monitored the development of higher-order capillary tube structures after 7 and 14 days of stimulation. WJ-MSCs (Figure 6A) and AD-MSCs (Figure 6B) cultured in the presence of micVEGF demonstrated capillary structure formation, whereas the condition without microspheres did not show such an effect.

CD31, also known as PECAM-1, is a specific marker of angiogenesis present in the mature endothelial cell membrane. As such, immunofluorescent antibody staining for CD31 was employed as a supplementary method of angiogenic differentiation confirmation. DAPI staining was also performed to analyze nuclei density and the arrangement of the WJ-MSCs and AD-MSCs. The results revealed the expression of PECAM-1 by WJ-MSCs (Figure 6C) and AD-MSCs (Figure 6D) only in the presence of micVEGF after 14 days, for both mesenchymal stem cell types. 

## 3. Discussion

Bone regeneration is an intricate process in which mesenchymal stem cells’ behavior is regulated by several environmental stimuli. Additionally, bone tissue survival exclusively relies on the development of a vascular network to secure all the necessary nutrients to retain homeostasis [32]. A crucial aspect for any potential drug delivery system is its ability to preserve the bioactivity of the loaded growth factors and avoid premature release of its contents [33]. Gelatin-based microspheres, although depicting great biological response and resorption capabilities, are weighed down by their rapid degradation rate, hindering their applicability as effective GF carriers [29]. Nguyen et al. [20], who studied the effect of varying methacrylation degrees on the stabilization of gelatin microspheres after photopolymerization, reported on a prolonged degradation rate up to 120 h, which is still considered very fast to replicate the gradual release profile required to properly direct the formation of neovascularized bone [34].

This study demonstrates that compositional tuning of gelatin–PVA hybrid microspheres enables modulation of degradation and growth factor release kinetics, resulting in sustained osteogenic and angiogenic differentiation of multiple MSC populations. Although PVA-based delivery systems are widely used, relatively few studies have directly examined how PVA content or crosslinking density influences both the release kinetics and the biological response of encapsulated cells. For example, PVA microgels with varying crosslinking densities significantly impacted the degradation rate, MSC viability, migration, and osteogenic differentiation as measured by ALP, calcium deposition, and *Runx2*/*OPN* gene expression [35]. Moreover, hydrogel studies show that higher PVA concentration tends to reduce porosity and water uptake, thereby modulating drug release [36]. Here, by tuning the PVA concentration in the developed gelatin–PVA microspheres, we aim to control growth factor release and evaluate how this tuning affects both osteogenic and angiogenic differentiation in different MSC types, thereby providing a more in-depth assessment of their regenerative potential.

The observed positive correlation between PVA concentration and microsphere diameter is consistent with prior reports showing that polymer concentration is a major determinant of droplet formation in emulsion and microfluidic processes [37]. Higher PVA content increases the viscosity and polymer chain entanglement of the dispersed aqueous phase, which reduces droplet breakup under shear and therefore produces larger droplets that solidify into larger microspheres. In addition, denser PVA layers at the oil–water interface can alter coalescence and interfacial stabilization, and residual PVA associated with particle surfaces can further modify apparent particle size and surface morphology [38]. Changes in PVA content have also been shown to slow release kinetics, likely by reducing pore size and water uptake, and to influence cell–matrix interactions [39]. Stiffer/denser PVA networks typically decrease diffusion but potentially enhance mechanical stability for long-term delivery. The observed lower cumulative release of BMP-2 and VEGF compared to BSA is consistent with the literature reports on gelatin-based carriers, where specific protein–matrix interactions and crosslinking density strongly affect retention and release kinetics. For instance, BMP-2 release from crosslinked gelatin microparticles exhibits minimal burst and slow sustained release that depends on the extent of crosslinking and binding to the matrix rather than simple diffusion [30]. Similarly, VEGF release is governed by molecular size, charge, and complexation with gelatin networks [40]. Growth factors bound to genipin-crosslinked gelatin microspheres have been reported to show incomplete release in the absence of enzymatic degradation, highlighting the role of affinity effects [29]. Broader reviews confirm that non-covalent interactions between growth factors and hydrogel matrices can dominate release behavior, explaining why model proteins like BSA, which lack specific binding domains, are released more readily [41].

The degradation and sustained-release behavior of the Gel–PVA microspheres can be directly correlated to differences in effective crosslinking density arising from variations in polymer composition. Glutaraldehyde covalently crosslinks gelatin chains, forming a stable network whose swelling, degradation, and transport properties depend on crosslinking density. Increasing PVA content enhances the density of this network through hydrogen bonding and physical chain entanglement, even in the absence of additional covalent bonds. This denser polymeric architecture restricts water penetration, reduces gelatin chain mobility, and limits pore enlargement during degradation, ultimately leading to slower mass loss and reduced diffusion of entrapped proteins. Similar effects of glutaraldehyde-mediated crosslinking density on gelatin hydrogel stability and transport behavior have been previously reported [42]. The experimentally observed lower degradation rate and sustained-release profile of the Gel–PVA10% microspheres are therefore attributed to the combined effects of covalent gelatin crosslinking and compositional tuning of the double gelatin–PVA network.

Microsphere size and size distribution are well-recognized determinants of controlled release performance since they affect the surface-area-to-volume ratio, diffusion path lengths, and drug distribution within the polymer matrix. Recent reviews have highlighted that particle size and uniformity are among the critical formulation parameters affecting encapsulation efficiency and release kinetics in particulate carriers, with smaller and more narrowly distributed particles generally exhibiting faster diffusion-controlled release due to a higher surface area relative to larger particles with broader distributions [43].

Moreover, formulation variables such as polymer ratios and emulsification parameters can alter size distributions and thereby modulate both protein loading and subsequent release profiles, as demonstrated in recent studies [44]. At the microstructural level, how drug molecules and domains are distributed within individual microspheres can also profoundly affect release behavior, particularly early burst and sustained phases, since drugs localized near the particle surface are released more rapidly than drug domains deeply embedded within the matrix [45]. In the developed system, although mean particle sizes were similar across Gel–PVA formulations, differences in size distribution and network density contribute to the observed release trends, with the denser networks and thus narrower effective diffusion paths of Gel–PVA10% retarding protein release over time.

All microspheres indicated a round shape morphology, which is a typical characteristic of the water-in-oil emulsion technique [46]. However, the inclusion of PVA appeared to affect the external surface roughness of the microspheres. This property of PVA, when used as a microsphere ingredient, has also been previously described [47] in PVA–nano-hydroxyapatite microspheres of incrementally increasing concentrations of the inorganic phase.

Degradation behavior is a key determinant of drug delivery performance, as it affects the structural stability of the carrier and the kinetics of the therapeutic release under physiological conditions [48]. In this context, the incorporation of PVA into gelatin-based microspheres appears to slow degradation, likely by reinforcing the hydrogel network through enhanced polymer chain entanglement and hydrogen bonding [49]. This stabilization not only supports slow and long-term release but may also preserve the bioactivity of sensitive proteins over time.

Differences in loading capacity across the formulations suggest that matrix composition plays a critical role in protein entrapment efficiency. Higher PVA content may create a more favorable environment for passive adsorption by reducing pore size and limiting premature diffusion, thereby increasing the effective payload. This aligns with previous reports indicating that denser polymer matrices can improve drug retention in hydrogel-based systems [50,51].

The incorporation of PVA into gelatin microspheres effectively modulated release kinetics, resulting in slower and more sustained protein delivery. This tunable behavior is particularly beneficial in the context of bone regeneration, where sequential and sustained exposure to multiple growth factors is essential for coordinated osteogenesis and angiogenesis. Compared to other reported systems, the current Gel–PVA10% microspheres demonstrated reduced initial burst release and longer-lasting release profiles. The low cumulative release of <10% reflects the dense polymeric network and reduced diffusion from the Gel–PVA10% formulation, supporting its suitability for long-term localized delivery rather than rapid depletion. For example, gelatin-based carriers reviewed in [52] typically release over 50% of their cargo within the first 48 h, whereas the developed formulation retained most of its payload beyond this time frame. It is important to note that low cumulative release percentages in vitro do not necessarily preclude biological efficacy, as controlled delivery systems are designed to provide sustained and localized presentation of growth factors that can maintain effective signaling over prolonged periods rather than releasing high amounts early on [53]. Unlike PLGA/PLLA microspheres that often exhibit high burst release and require organic solvents [54], the Gel–PVA system enables protein loading through mild aqueous adsorption while maintaining prolonged retention. Similarly, while hydrogel-integrated delivery platforms [55] and chitosan-based microspheres [56] have shown co-delivery capabilities, they are either fabrication-intensive or exhibit higher burst and shorter duration. The ability to tune release kinetics by varying the PVA content, coupled with high compatibility with multiple stem cell types, positions this system as an adaptable platform for tissue regeneration applications.

The reduced burst release of BMP-2 and VEGF compared to BSA reflects their stronger interactions with the gelatin–PVA matrix. Growth factors exhibit higher binding affinity and lower diffusivity than BSA, leading to improved retention and reduced initial loss [57]. Additionally, the observed difference in loading capacity between VEGF and BMP-2 may be attributed to the different initial loading concentrations, as BMP-2 was loaded at 0.2 μg/mL, while VEGF was loaded at 0.1 μg/mL, as well as their molecular characteristics, such as size, charge, and hydrophilicity. The size of VEGF and its favorable structural characteristics may enhance its passive adsorption or entrapment within the microsphere matrix [57], particularly in systems without covalent immobilization [58]. Previous studies have shown that growth factors incorporated into crosslinked gelatin carriers remain largely matrix-bound under non-enzymatic conditions and are primarily released upon matrix biodegradation [20]. Importantly, gelatin hydrogels have been reported to preserve growth factor bioactivity during sustained delivery [59]. In agreement with these findings, the released fraction in the present study remained biologically functional, as demonstrated by the enhanced osteogenic and angiogenic responses. Future studies under enzymatic conditions would clarify the long-term release behavior.

The biocompatibility of drug delivery systems is a critical factor affecting their translational potential, particularly when intended for local implantation or injection. In this study, all microsphere formulations exhibited good cytocompatibility up to 1 mg/mL, suggesting minimal cytotoxicity associated with the components at relevant doses. Selecting a working concentration of 0.5 mg/mL therefore represents a balanced selection that maximizes content delivery potential while avoiding cell stress or reduced viability. Importantly, this concentration supported fibroblast adhesion, suggesting favorable surface properties for cell–material interaction, which is essential for tissue integration and signal transduction in regenerative applications.

The process of osteogenesis is driven by a cascade of biological processes initiated by the recruitment of BM-MSCs to bone remodeling sites, which is accompanied by various intricate events, including cell proliferation, formation of an extracellular matrix, and differentiation towards specific lineages [60]. Osteogenic differentiation of BM-MSCs has been reported to be affected by the regulated release of BMP-2 at various stages of de novo bone formation [61]. The presence of micBMP2 did not appear to negatively affect cell viability, while data from all osteogenic-related assays, including the ALP activity, calcium deposition, and collagen secretion, disclosed significant upregulation of these markers compared to the non-treated control condition. These findings align with a recent study [62] that demonstrated that BMP-2 enhances ALP expression and mineralization via a Wnt autocrine loop, highlighting the interplay between BMP and Wnt signaling pathways in osteoblast differentiation. The sustained release system developed in this study offers a potentially more controlled and prolonged activation of these pathways, which could be advantageous in bone tissue engineering.

Angiogenesis is an important physiological process required for bone healing of injured tissues, as it brings oxygen and nutrients to the injury site. Microenvironment factors, including angiogenic growth factors, proangiogenic factors, physical forces, extracellular matrix, and hypoxia, regulate angiogenesis [63]. Undifferentiated mesenchymal stem cells typically exhibit a fibroblast-like appearance, whereas endothelial cells (ECs) display a characteristic cobblestone organization morphology [64]. The transition of the MSCs towards the capillary configuration was apparent even by day 7 in the micVEGF-treated cells, signifying the effect of incremental VEGF release from the microspheres. Previous studies have reported that when MSCs are exposed to even low dosages of VEGF-A, they tend to form clusters of cobblestone-shaped cells as an indicator of the transition to mature endothelial morphology [65]. The effect of micVEGF was profound on the expression of CD31 in both mesenchymal stem cell types, whereas the control condition showed a lack of activation. CD31 plays an important role as a modulator of the endothelial cell-to-cell contact in angiogenesis [66]. While single-dose versus sustained VEGF delivery comparisons would further elucidate the advantages of controlled release, the present in vitro assay was designed to confirm VEGF bioactivity following encapsulation and release, a prerequisite for such comparative studies.

Unlike other previously reported gelatin- or PVA-based delivery systems that rely on chemical conjugation of high growth factor doses or rapid release profiles, the developed Gel–PVA microspheres achieve sustained retention and bioactivity through compositional tuning. This approach enables long-term, localized delivery of osteogenic and angiogenic cues while minimizing burst release and potential side effects, rendering this system a versatile platform for vascularized bone regeneration. Although gelatin- and PVA-based delivery systems have been explored, recent studies emphasize chemical modification, functionalization, or complex stimuli-responsive designs to achieve controlled release. In contrast, the present work demonstrates that solely compositional tuning, under fixed processing and crosslinking conditions, provides a predictable system to regulate microsphere degradation and protein release kinetics. By tuning the PVA concentration, we were able to modulate the degradation rate and release kinetics, ultimately identifying the Gel–PVA10% formulation as optimal in terms of protein retention, cytocompatibility, and biological activity. This approach enables a clearer interpretation of how physical network density governs protein retention and diffusion. Such insights are particularly relevant for translational and scalable formulation development, where simplicity, reproducibility, and compatibility are often prioritized over chemical complexity. This system supported both osteogenic and angiogenic differentiation across multiple MSC types, suggesting its applicability in bone tissue engineering.

This study addressed the challenge of delivering osteogenic and angiogenic cues in a controlled and sustained manner by developing Gel–PVA microspheres of different compositions, capable of delivering BMP-2 and VEGF. Unlike other previously reported Gel–PVA delivery systems that rely on chemical conjugation, surface functionalization, or complex crosslinking strategies to achieve sustained release, the present work demonstrates that compositional tuning of PVA content blended with a constant gelatin concentration is sufficient to modulate growth factor retention and release kinetics. Although BMP-2 and VEGF were delivered separately in this study to highlight their individual effects, the modular nature of the Gel–PVA microspheres makes them suitable for co-loading strategies. Future studies may explore co-loading scenarios, protein immobilization stability, and integration within 3D scaffolds, while the validation of in vivo performance and long-term efficacy would enhance the relevance of this study for clinical translation.

## 4. Conclusions

In this study, we explored the properties of Gel–PVA microspheres as a potential delivery system for BMP-2 and VEGF and evaluated their capability to elicit osteogenic and angiogenic responses in BM-MSCs, WJ-MSCs, and AD-MSCs, respectively. Among the three different concentrations examined, the Gel–PVA10% formulation exhibited the highest protein loading capacity and encapsulation efficiency, the lowest cumulative release rate, and excellent cytocompatibility at concentrations of up to 0.5 mg/mL. Based on the combined physicochemical and biological performance, Gel–PVA10% microspheres at 0.5 mg/mL were selected for the subsequent assessment of growth factor bioactivity. The alkaline phosphatase activity, collagen secretion, and calcium mineralization were significantly upregulated in BM-MSCs treated with BMP-2-loaded microspheres, while VEGF-loaded microspheres enhanced tubular formation and PECAM-1 expression in WJ-MSCs and AD-MSCs. These findings demonstrate that the Gel–PVA10% formulation provides a stable, biomimetic platform for the controlled delivery of osteogenic and angiogenic cues, highlighting its promise for vascularized bone tissue regeneration.

## 5. Materials and Methods

### 5.1. Preparation of Gelatin–PVA Microspheres

**Figure 7:**
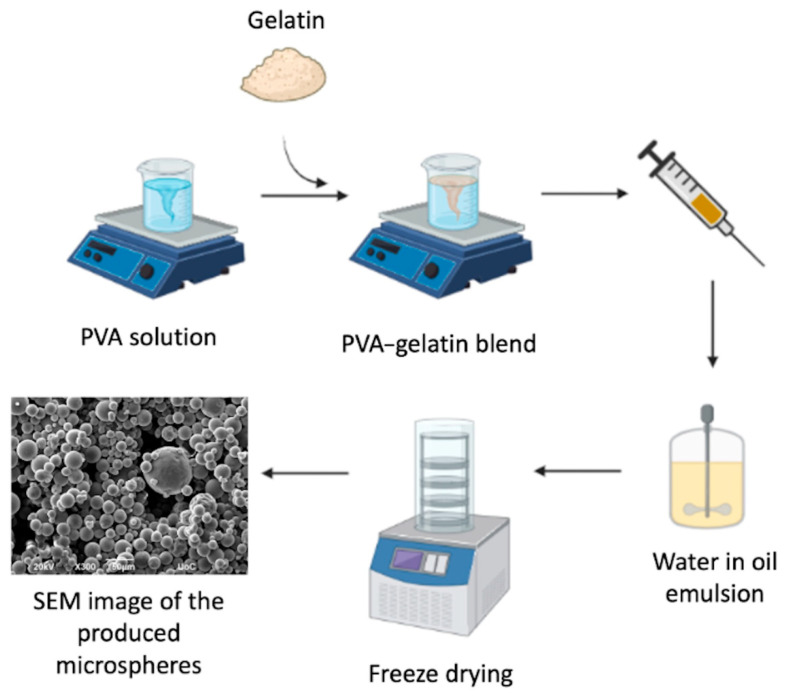
Schematic representation of the production steps of Gel–PVA microspheres.

For the preparation of the microspheres, we followed a modified protocol of the emulsion crosslinking technique for gelatin-based microspheres [67]. Briefly, aqueous PVA solutions of 0%, 5%, and 10% *w*/*v* were allowed to stir at 90 °C for 8 h. Gelatin at a concentration at 10% *w*/*v* was added to each solution, and the mixtures were allowed to homogenize for up to 1 h. Subsequently, the mixtures were added dropwise through a syringe to sunflower oil, which was preheated at 50 °C, to create a water-in-oil emulsion, and then 1 mL of surfactant Span 80 was poured to stabilize the emulsion. The solution was stirred at 1000 rpm at 60 °C for 15 min, and then stirring resumed at temperatures close to 0 °C for 1.5 h under constant stirring to promote gelatin gelation [68]. At this stage, glutaraldehyde was added at a final concentration of 0.025% *v*/*v*. Glutaraldehyde acts as a bifunctional crosslinking agent that primarily reacts with free amine groups of gelatin through Schiff base formation, resulting in covalent inter- and intramolecular crosslinks within the gelatin network. Under the applied reaction conditions, polyvinyl alcohol does not participate in covalent crosslinking with glutaraldehyde; however, it contributes to network stabilization through extensive hydrogen bonding and polymer chain entanglement with gelatin. Therefore, although the glutaraldehyde concentration was kept constant across all formulations, variations in PVA content effectively modulate the overall network density of the microspheres. After 1.5 h, 30 mL of pure acetone was added and stirred for 40 min at 0 °C. Gelatin microspheres were collected by centrifugation at 3500 rpm and washed with acetone. Centrifugation products were freeze-dried for 48 h at −20 °C. The workflow is schematically shown in Figure 7, while the compositions of the hybrid microspheres and their abbreviations are presented in Table 1.

### 5.2. Physicochemical Characterization of Gel–PVA Microspheres

#### 5.2.1. Imaging of Microspheres by SEM

The size and shape of unloaded gelatin microspheres were observed via SEM. Particle size distributions were determined from SEM images acquired at ×100 magnification to ensure a representative sampling area, while higher-magnification images (×400 or ×500) were used for qualitative assessment of microsphere surface morphology. Samples for SEM analysis were prepared by gold sputtering the microsphere powders, and their size was measured from the investigation of SEM images using ImageJ software version 1.54k (National Institutes of Health, Bethesda, MD, USA). To determine the frequency distribution, a Gaussian curve (normal distribution) was plotted by means of GraphPad Prism version 8 (GraphPad Software, San Diego, CA, USA). The values are reported as the mean ± standard deviation.

#### 5.2.2. FTIR Spectra Evaluation

The FTIR analysis results of the different microsphere compositions were recorded using a Nicolet 6700 optical spectrometer (ThermoFisher Scientific, Madison, WI, USA) within the region of 400–4000 cm^−1^. The spectra data were collected, and then the numerical values were transferred to the Origin software version 10.2 for graphical representation.

#### 5.2.3. Degradation Studies

To determine the microsphere degradation of the different compositions, 10 mg of unloaded microspheres (W_0_) was incubated in 2 mL of phosphate buffer saline (PBS) at room temperature for 48 h before commencing the degradation studies. After incubation in PBS, the microspheres were washed with deionized water, freeze-dried at −20 °C for 24 h, and then weighed (W_e_). The % of degradation of each sample was calculated through the following equation:

% degradation = [(W_0_ − W_e_)/W_0_] × 100%,

where W_0_ is the weight of unloaded dry microspheres; W_e_ is the weight of unloaded dehydrated microspheres

#### 5.2.4. In Vitro Protein Absorption and Release Study

The capacity of microspheres to incorporate biological molecules, as well as their ability to release them in a sustainable manner, was assessed using bovine serum albumin (BSA) as the model protein. Gel–PVA microspheres were loaded with BSA via physical adsorption, by mixing 12 mL of a 4 mg/mL BSA solution in PBS with 30 mg of dried microspheres at 37 °C for 48 h. The loaded microspheres were recovered by centrifugation and dried in a desiccator at room temperature. The concentration of the residual BSA in the supernatant was determined by the bicinchoninic acid (BCA) assay for protein quantitation. The % of BSA encapsulation efficiency (%EE) and % of microsphere loading capacity (%LC) were determined using the following equations:

% BSA EE = (Initial weight of BSA − Weight of BSA in supernatant)/(Initial weight of BSA) × 100%

% mic LC = (Initial weight of BSA − Weight of BSA in supernatant)/(Dry weight of microspheres) × 100%.

Encapsulation efficiency reflects the percentage of drug that was successfully entrapped in a drug carrier. Loading capacity is the amount of drug loaded per unit of particle weight.

The release kinetics of the BSA model protein were quantified according to a protocol previously described [69]. Briefly, 10 mg of gelatin microspheres was placed in 2 mL of PBS solution, and then incubated at 37 °C in a thermomixer (Eppendorf Thermomixer 5350 Mixer) at 300 rpm. At predetermined time intervals, the supernatant was collected after centrifugation at 2000 rpm for 2 min and replaced with the same volume of fresh PBS. The amount of BSA released from the microspheres was determined by BCA assay and expressed as a percentage of the total BSA that was entrapped in the samples. Cumulative protein release was calculated by summing the absolute amount of BSA released at each sampling time and normalizing this value to the total amount of BSA initially encapsulated in the microspheres. Released protein concentration represents the absolute amount of BSA present in the release medium at each time point, and is therefore not directly proportional to the corresponding % of cumulative release.

### 5.3. In Vitro Cytocompatibility Assessment of the Microspheres

The fibroblast-like cell line L929 (DSMZ Braunschweig, Germany, ACC-2) was used to assess cell viability and proliferation. The cells were cultured in RPMI culture medium (Biosera, Cholet, France), supplemented with 10% fetal bovine serum (FBS) 10% *v*/*v* (Gibco, Paisley, UK), 50 IU/mL penicillin (Sigma-Aldrich, St. Louis, MO, USA), and 50 g/mL streptomycin (Sigma-Aldrich, St. Louis, MO, USA)] in a 5% CO_2_ ThermoFisher incubator (Waltham, MA, USA) at 37 °C.

Cytotoxicity was evaluated according to ISO 10993-5:2009 standard ‘Biological evaluation of medical devices—Part 5: Tests for in vitro cytotoxicity’ based on Wallin’s guidance [70]. Cell viability was measured by the PrestoBlue^®^ viability assay (Invitrogen, Carlsbad, CA, USA), based on the manufacturer’s instructions. Briefly, 10^4^ cells per well were seeded in 96-well plates for 24 h. The next day, the culture medium was replaced with medium containing five different microsphere concentrations of 0.125, 0.25, 0.5, 1, and 2 mg/mL to determine the most biocompatible concentration. Cell viability was measured after 24 and 48 h. The measurements were performed by using a BioTek Synergy HTX microplate reader (Winooski, Vermont, VT, USA), and the absorbance was measured at 570 and 600 nm. The control refers to cells cultured on the surface of the tissue culture-treated polystyrene (TCPS). Data represent means ± standard deviation of quadruples.

SEM observation was performed for the illustration of adhesion and morphology after 7 and 14 days in culture. In this regard, 5 × 10^3^ cells per well were seeded in 96-well plates on day 0, and on day 1, the culture medium was replaced with medium containing 0.5 mg/mL Gel–PVA10% microspheres. Cell viability was measured at days 3, 7, and 14. On day 7 and day 14, samples were rinsed three times with PBS, fixed with 4% *v*/*v* paraformaldehyde for 20 min, and dehydrated in increasing concentrations (30–100% *v*/*v*) of ethanol. The samples were then dried in a critical point dryer Baltec CPD 030 (Balzers, Liechtenstein), sputter-coated with a 20 nm thick layer of gold by means of Baltec SCD 050 (Balzers, Liechtenstein), and observed under SEM at an accelerating voltage of 15 kV.

### 5.4. Loading of the Gel–PVA10% Microspheres with BMP-2 and VEGF

Recombinant Human VEGF 165 (*E. coli* expressed) protein was purchased from R&D Systems, Minneapolis, MN, USA, while the recombinant human bone morphogenetic protein 2 (rhBMP-2) was purchased from Prospecbio International (Ness-Ziona, Israel). For the investigation of osteogenic differentiation, the Gel–PVA10% microspheres were immersed in 0.2 μg/mL BMP-2 in PBS solution and maintained at 37 °C for 2 h on an orbital shaker to ensure continuous mixing. For endothelization experiments, Gel–PVA10% microspheres were placed in microcentrifuge tubes containing 0.1 μg/mL VEGF in PBS at 4 °C for 12 h. The BMP-2 and VEGF loading concentrations were selected based on reported effective ranges and the sustained release profile of the developed system [71,72]. BMP-2 has been shown to induce osteogenic differentiation of mesenchymal stem cells at concentrations as low as ~10 ng/mL, with enhanced effects observed up to ~50–100 ng/mL, while VEGF has been reported to promote angiogenic responses at concentrations in the range of ~1–50 ng/mL [73,74]. Notably, synergistic effects between BMP-2 and VEGF have been reported, even at low concentrations, emphasizing the importance of controlled presentation rather than high-dose delivery [73]. In this study, although the cumulative release of growth factors from Gel–PVA10% microspheres was low, the sustained release over time is expected to maintain local concentrations within biologically relevant ranges, which is consistent with previous reports highlighting the importance of prolonged exposure for effective osteogenic and angiogenic differentiation [75].

### 5.5. In Vitro GF Absorption and Release Study

The % of encapsulation efficiency and the % of microsphere loading capacity of the micBMP2 and the micVEGF were determined in a manner similar to that described in Section 5.2.4. The amount of the GF released from the microspheres was evaluated via enzyme-linked immunosorbent assay (ELISA), according to the manufacturer’s protocol, and expressed as a percentage of the total GF that was entrapped in the samples. For the determination of VEGF levels in the supernatants, VEGFA sandwich ELISA capture (855.920.000, Diaclone, Besançon, France) and detection (855.930.000, Diaclone) antibodies were employed according to the manufacturer’s protocol. As for BMP-2, the quantity released was quantified using a BMP-2 ELISA kit ab119581 (Abcam, Cambridge, UK) following standard procedures. Four replicates were performed for each of these experiments.

### 5.6. Osteogenic Response of HUMAN BM-MSCs in the Presence of BMP-2-Loaded Gel–PVA Microspheres

#### 5.6.1. Isolation and Culture of Human BM-MSCs

Bone marrow samples were taken from adult donors undergoing hip replacement surgery. Written informed consent was obtained from the donors, and institutional ethics approval was granted according to the Bioethics Committee of the University of Crete, protocol No. 26-05-2010/3910. The median age of the patients was 45 years. BM aspirate was mixed with 3000 units of preservative-free heparin and diluted at 1:1 (*v*/*v*) with phosphate-buffered saline (PBS) (Sigma-Aldrich, Taufkirchen, Germany). BM mononuclear cells (BM-MNC) were collected after gradient centrifugation in Histopaque-1077 (Sigma-Aldrich, Taufkirchen, Germany) and cultured in alpha-modified Eagle’s medium (alpha-MEM) from Gibco (Paisley, UK), supplemented with 2 mM L-glutamine, 100 U/mL penicillin/streptomycin (Sigma-Aldrich, Taufkirchen, Germany), and 10% fetal bovine serum (FBS) (Sigma-Aldrich, Taufkirchen, Germany) at 37 °C, in a 5% CO_2_ fully humidified atmosphere, as described elsewhere [1]. Culture medium was replaced twice a week; when adherent BM-MSCs formed visible colonies (after 10–14 d of culture), they were removed using 0.25% trypsin-1 mM EDTA (Gibco, Paisley, UK) and re-seeded at a density of 2 × 10^3^ cells/cm^2^. When BM-MSCs reached 90% confluency, they were harvested using 0.25% trypsin-EDTA and re-seeded at a concentration of 2 × 10^3^ cells/cm^2^ and further expanded for a total of three passages. For viability and differentiation assays, 10^4^ cells per well were seeded onto 96-well plates. The medium was replaced every three days. For differentiation experiments, the primary culture medium was supplemented with 50 μg/mL l-ascorbic acid, 10 mM β-glycerophosphate, and 10 nM dexamethasone. Freshly prepared Gel–PVA10% loaded with 0.2 μg/mL BMP-2 solution (micBMP2) was added to a total concentration of 0.5 mg/mL osteogenic media. All experiments were carried out using cells at passage numbers 2 and 3.

#### 5.6.2. Alkaline Phosphatase (ALP) Activity of BM-MSCs

ALP enzymatic activity was quantified to assess the osteogenic response of BM-MSCs cultured in the presence or absence of micBMP2. The cells were maintained in osteogenic differentiation medium—consisting of basal medium supplemented with 10 nM dexamethasone, 50 μg/mL ascorbic acid, and 0.1 mM β-glycerophosphate—and harvested after 3, 7, and 14 days of culture. At each time point, the cells were detached using trypsin-EDTA, collected by centrifugation, and lysed in 100 μL of lysis buffer (0.1% Triton X-100 in 50 mM Tris-HCl, pH 10.5). Cell lysates were subjected to two freeze–thaw cycles between −80 °C and room temperature to ensure complete cell disruption. An amount of 100 μL of p-nitrophenyl phosphate (pNPP; 2 mg/mL), prepared in 50 mM Tris-HCl buffer (pH 10.5) containing 2 mM MgCl_2_, was added to each sample and incubated at 37 °C for 1 h. The enzymatic reaction was terminated by the addition of 50 μL of 1 N NaOH. Absorbance was recorded at 405 nm using a BioTek Synergy HTX microplate reader (Winooski, Vermont, VT, USA), and ALP activity was calculated based on a para-nitrophenol standard curve. Enzymatic activity values were normalized to total protein content as determined by the Bradford assay. All experimental conditions were tested in triplicate across at least two independent experiments (*n* = 6).

#### 5.6.3. Determination of the Calcium Biomineralization by BM-MSCs

The extent of extracellular matrix mineralization was evaluated using Alizarin Red staining. BM-MSC cultures maintained in osteogenic medium for 7, 14, and 21 days were rinsed twice with PBS and fixed with 4% paraformaldehyde for 20 min at room temperature. Calcium-rich deposits were stained with Alizarin Red S following established protocols. Stained cultures were visualized using an inverted light microscope (Axiovert 200, Carl Zeiss, Berlin, Germany), and images were acquired using a ProgRes CF scan camera and ProgRes Capture Pro software version 2.8.8 (Jenoptik Optical Systems GmbH, Berlin, Germany).

For quantitative analysis, bound dye was eluted using cetylpyridinium chloride (CPC). Briefly, 300 μL of 10% (*w*/*v*) CPC prepared in 10 mM sodium phosphate buffer was added to each sample and incubated for 1 h under continuous agitation. The absorbance of the extracted dye was measured at 550 nm using a BioTek Synergy HTX microplate reader (Winooski, Vermont, VT, USA). Mineralization levels were normalized to cellular protein content determined by the Bradford assay. Each condition was analyzed in triplicate in at least two independent experiments (*n* = 6).

#### 5.6.4. Determination of the Produced Extracellular Collagen by the BM-MSCs

Total collagen secretion by BM-MSCs was determined using the Sirius Red dye assay [76]. At the designated time points, 25 μL of culture supernatant was diluted to a final volume of 100 μL and mixed with 1 mL of 0.1% (*w*/*v*) Sirius Red solution. Samples were incubated for 30 min at room temperature to allow collagen–dye complex formation. The mixtures were then centrifuged at 15,000× *g* for 15 min, and the resulting pellets were washed with 0.1 N HCl to remove unbound dye. After a second centrifugation step at 15,000× *g* for 15 min, the collagen-bound dye was solubilized in 500 μL of 0.5 N NaOH. Absorbance was measured at 530 nm using a BioTek Synergy HTX microplate reader (Winooski, Vermont, VT, USA). Collagen concentrations were calculated based on a calibration curve generated with collagen type I standards. All measurements were performed in triplicate across at least two independent experiments (*n* = 6).

### 5.7. Angiogenic Response of Human WJ-MSCs and Human AD-MSCs in the Presence of VEGF-Loaded Gelatin–PVA Microspheres

#### 5.7.1. Isolation and Culture of Human WJ-MSCs

Wharton’s jelly mesenchymal stem cell (WJ-MSC) isolation and culture were performed as described previously [77]. Briefly, umbilical cord was collected after written informed consent was obtained from the donors, according to the Bioethics Committee of the University of Crete, approved protocol No. 1724/14-02-2012. Wharton’s jelly from the inner lining of the cord was chopped and plated. Adherent MSCs grew from the WJ tissue within two weeks. WJ-MSCs were then expanded in alpha-MEM cell culture medium supplemented with 10% FBS, 2 mM L-glutamine, and 100 IU/mL penicillin/streptomycin (complete alpha-MEM) (all from Invitrogen, Carlsbad, CA, USA). Subsequently, the cells were harvested using 0.25% trypsin-EDTA (Gibco) and a subculture thereafter at a plating density of 10^3^ cells/cm^2^ for 4 passages.

WJ-MSCs and AD-MSCs (Lonza, Basel, Switzerland) were cultured in alpha-MEM medium supplemented with 10% fetal bovine serum (FBS), 2 mM L-glutamine, 100 μg/mL penicillin/streptomycin, and 2.5 μg/mL amphotericin (fungizone) in a humidified incubator at 37 °C and 5% CO_2_. The cells were harvested using trypsin/EDTA, with 10^4^ cells per well used for all experiments, and then the cells were seeded onto the 96-well plates. The medium was replaced every three days. Freshly prepared Gel–PVA10% loaded with 0.1 μg/mL VEGF solution (micVEGF) was added to a total concentration of 0.5 mg/mL osteogenic media. All experiments were carried out using cell passages 2 to 4.

#### 5.7.2. Visualization of Cell Morphology in the Presence of micVEGF

The monitoring of morphological changes in two mesenchymal stem cell lines, WJ-MSCs and AD-MSCs, in the presence of micVEGF, was conducted using an inverted optical microscope (Axiovert 200, Carl Zeiss, Berlin, Germany). Photographs were taken by employing a ProgRes CF scan camera and its compatible software ProgRes Capture Pro version 2.8.8 (Jenoptik Optical Systems GmbH, Berlin, Germany). Optical microscopy images were obtained for each cell type to evaluate the cell morphology regulation in the presence of micVEGF after 7 and 14 days in culture, and were then compared to the non-treated control cultures.

#### 5.7.3. Immunofluorescent Staining of Platelet Endothelial Cell Adhesion Molecule 1 (PECAM-1)

The expression of characteristic endothelial cell marker PECAM-1 was investigated after the WJ-MSCs and AD-MSCs were cultured for 14 days in the presence and absence of micVEGF. The cells were washed three times in PBS and fixed in 4% paraformaldehyde in PBS for 20 min. 4′,6-diamidino-2-phenylindole (DAPI) staining was used to detect nuclei. The samples were treated with 300 nM for 15 min and then thoroughly washed with PBS. Subsequently, the cells were treated with blocking buffer (5% FBS/0.3% Triton™ X-100 in PBS) for 1 h. The CD31 (PECAM-1) (89C2) Mouse mAb (Alexa Fluor^®^ 647 Conjugate) antibody was diluted according to the manufacturer’s instructions and incubated overnight at 4 °C. After three washes with PBS, the substrates were dried and then examined using an inverted fluorescence microscope (Axiovert 200, Carl Zeiss, Berlin, Germany). Photographs were taken by employing a ProgRes CF scan camera and its compatible software ProgRes Capture Pro (Jenoptik Optical Systems GmbH, Berlin, Germany). All experiments were carried out using cell passages ranging between 2 and 4.

## Figures and Tables

**Table 1:** Sample abbreviations and compositions.

Abbreviation	Compositions
Gel–PVA0%	10% *w*/*v* gelatin, without PVA, crosslinked with 0.025% *v*/*v* glutaraldehyde
Gel–PVA5%	10% *w*/*v* gelatin and 5% *w*/*v* PVA crosslinked with 0.025% *v*/*v* glutaraldehyde
Gel–PVA10%	10% *w*/*v* gelatin and 10% *w*/*v* PVA crosslinked with 0.025% *v*/*v* glutaraldehyde
micBMP2	Gel–PVA10% crosslinked with 0.025% *v*/*v* glutaraldehyde and loaded with 0.2 μg/mL BMP-2
micVEGF	Gel–PVA10% crosslinked with 0.025% *v*/*v* glutaraldehyde and loaded with 0.1 μg/mL VEGF

## Data Availability

The dataset is available upon request from the authors.
